# Color translation from monoscopic photogrammetry +ID Methodology into a Polyjet final 3D printed facial prosthesis.

**DOI:** 10.12688/f1000research.111196.1

**Published:** 2022-05-27

**Authors:** Rodrigo Salazar-Gamarra, Andrés Cárdenas-Bocanegra, Uri Masch, Cícero André Da Costa Moraes, Rosemary Seelaus, Jorge Vicente Lopes Da Silva, Luciano Lauria Dib

**Affiliations:** 1 Norbert Wiener University - Digital Transformation Research Centre, Lima, 15046, Peru; 2 Plus Identity (+ID) Institute, São Paulo, 04057-000, Brazil; 3 Stratasys Latin America, São Paulo, 04514-041, Brazil; 4 Stratasys, Rehovot, 76100, Israel; 5 The Craniofacial Center, Department of Surgery, University of Illinois at Chicago, Chicago, 60612, USA; 6 Centro Tecnológico da Informação Renato Archer, Campinas, 13069-901, Brazil; 7 Paulista University, São Paulo, 04057-000, Brazil

**Keywords:** 3D printing, color, head and neck neoplasms, prosthesis design, maxillofacial prosthesis

## Abstract

**Background:** The artistic techniques necessary to fabricate facial prostheses mainly depend on individual skill and are not a resource easily reproduced. Digital technology has contributed to improved outcomes, often combining analog and new digital techniques in the same workflow.

**Methods:** This article aims to present an innovative workflow to produce a final colored 3D printed and facial prosthesis by UV-map color translation into colored resin 3D printing. A modified +ID Methodology was used to obtain 3D models with the calibrated 3D printable patient’s skin color. No hands-on physical molding, manual sculpture, or intrinsic silicone coloration was used.

**Results:** The outcome resulted in acceptable aesthetics, adaptation, and an approximate color match after extrinsic coloration. The patient reported good comfort and acceptance.

**Conclusions:** A direct resin 3D printed prosthesis may be a viable alternative, especially for rapid delivery as an immediate prosthesis or an option when there is no experienced anaplastogist to manufacture a conventional prosthesis.

## Introduction

The customized production of facial prostheses is a worldwide challenge due to multiple factors. Lack of formal education programs, a steep technical learning curve with associated high costs, the working time and labor-intensive nature of the work, lack of reliable supply chain for materials, and limited health system coverage are among the many challenges. Among the maxillofacial prosthodontics and anaplastology services, the most significant challenges from the fabrication perspective are the processes of molding, sculpture, and coloration reproduction. The technique-specific processes that mainly rely on individual artistic capabilities represent a scarce clinical resource. ^1^^,^^2^

In the past two decades, digital technology has been progressively overcoming some of these challenges being a source of innovation for techniques, methods, and biomaterials, combined with conventional methods for optimizing the efficiency and outcomes of facial prosthetic rehabilitation. ^3^^–^^8^ Some workflows have been proposed combining different hardware & software tools for a) data acquisition (CT scans, MRI, laser, structured light or photogrammetry scanners), b) 3D modeling resources (mirrored anatomy, digital manual sculpting, digital donors or digital libraries, etc.) and c) 3D manufacture methods like 3Dprinting (FDM, SLA, DLP, LCD, Polyjet, SLS, etc.) or CNC milling machines. ^7^^,^^9^^–^^12^

Due to the high cost of some of these resources, one of the recent trends is achieving digital results using more accessible resources. In that sense, the authors previously proposed a complete workflow for 3D accessible facial prosthesis, called Plus ID Methodology (+ID). It uses monoscopic photogrammetry from smartphone captures, open-source software, and low-cost 3D printers. ^3^^,^^7^

More recently, efforts about direct 3D printing of facial prosthetic materials and semi-final devices have been evolving. Physical and chemical optimization of the elastomeric material properties have been tested for suitable additive manufacture production for facial prosthetics. ^13^^,^^14^ Also, authors have published reports on 3D printed nasal and auricular prosthesis as semi-final devices in medical-grade silicone, multi-material consistency silicone, and flexible resin to produce interim prosthesis. However, enhancements and limitations were recognized in terms of technology, ideal characteristics of the materials proposed, biocompatibility, margin adaptation, and desired combined properties as one unique solution. ^5^^,^^6^^,^^15^

In a parallel line, the industry of resin 3D printing has been advancing in color 3D printing. VRML files allow carrying the voxel-color information from UV-Map JPG files in a common language for additive manufacturing, as CMYK works for 2D printing. The monoscopic photogrammetry 3D models obtained by +ID are suitable for calibration and export into VRML files. They adequately communicate with a specific resin 3D printer compatible with color 3D printing, like the Stratasys J750 ^®^.

The possibility of using color translation from accessible 3D scanning and computer graphic tools to overcome color reproduction with greater automation, and less manual work, is promising for a more productive and practical solution for future patients and professionals in facial prosthetics.

The purpose of this publication is to contribute an innovative methodology and multi-material color resin 3D printing technique as a proposed combined specific workflow of digital resources to obtain a resin full-color 3D printed facial prosthesis for an orbital prosthesis by full-color Polyjet 3D printing.

## Methods and results

A 56-year-old man from our PlusID Maxillofacial Prosthetics department at the Universidade Paulista (UNIP) was due to renew his old prosthesis and voluntarily agreed to participate in the present study after reviewing ethical considerations and signing the informed consent. The research was given ethics committee approval N° 2.509.955 by the ethics committee “Associacao Unificada Paulista De Ensino Renovado Objetivo” CAAE 83301517.7.0000.5512 regulated by the Brazilian Platform.

### Data acquisition

**Figure 1.**
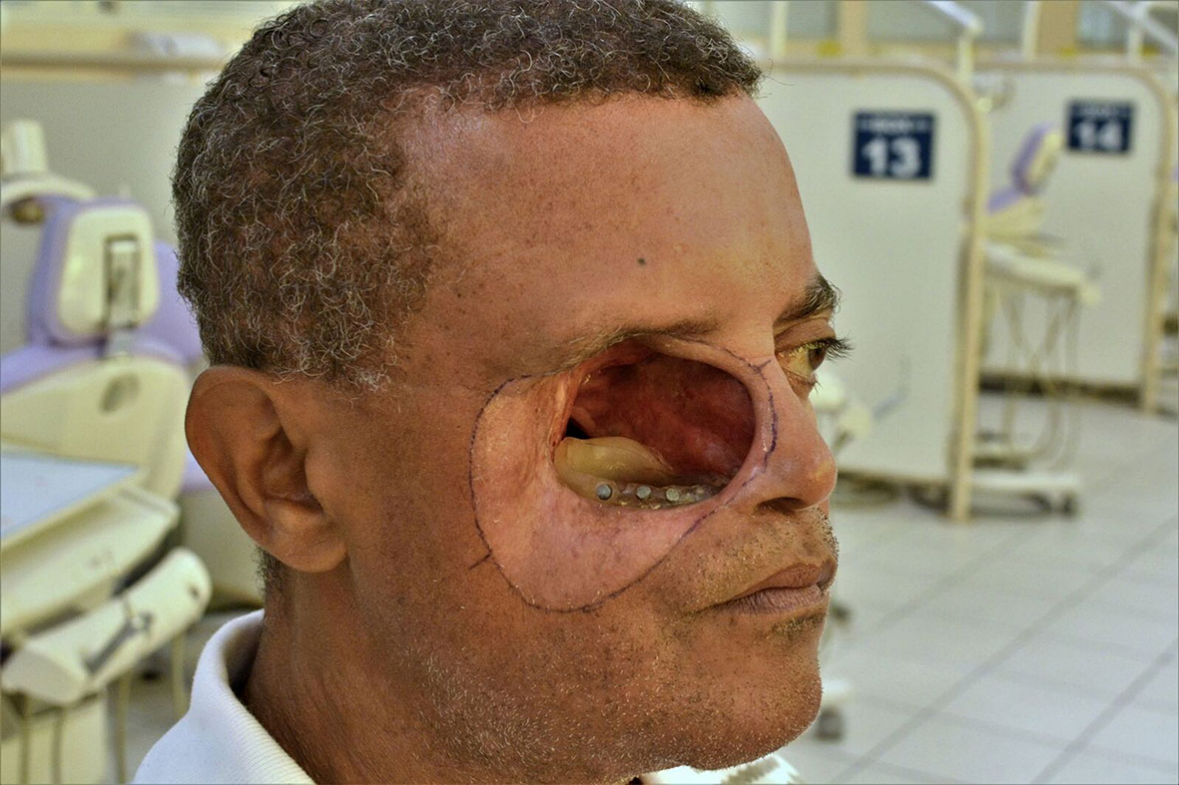
Clinical delineation of the limits of the process is determined before 3D photogrammetry capture. (Written informed consent for publication of the patient’s details and publication of the identifiable image in Figure 1 was obtained from the patient).

**Figure 2.**
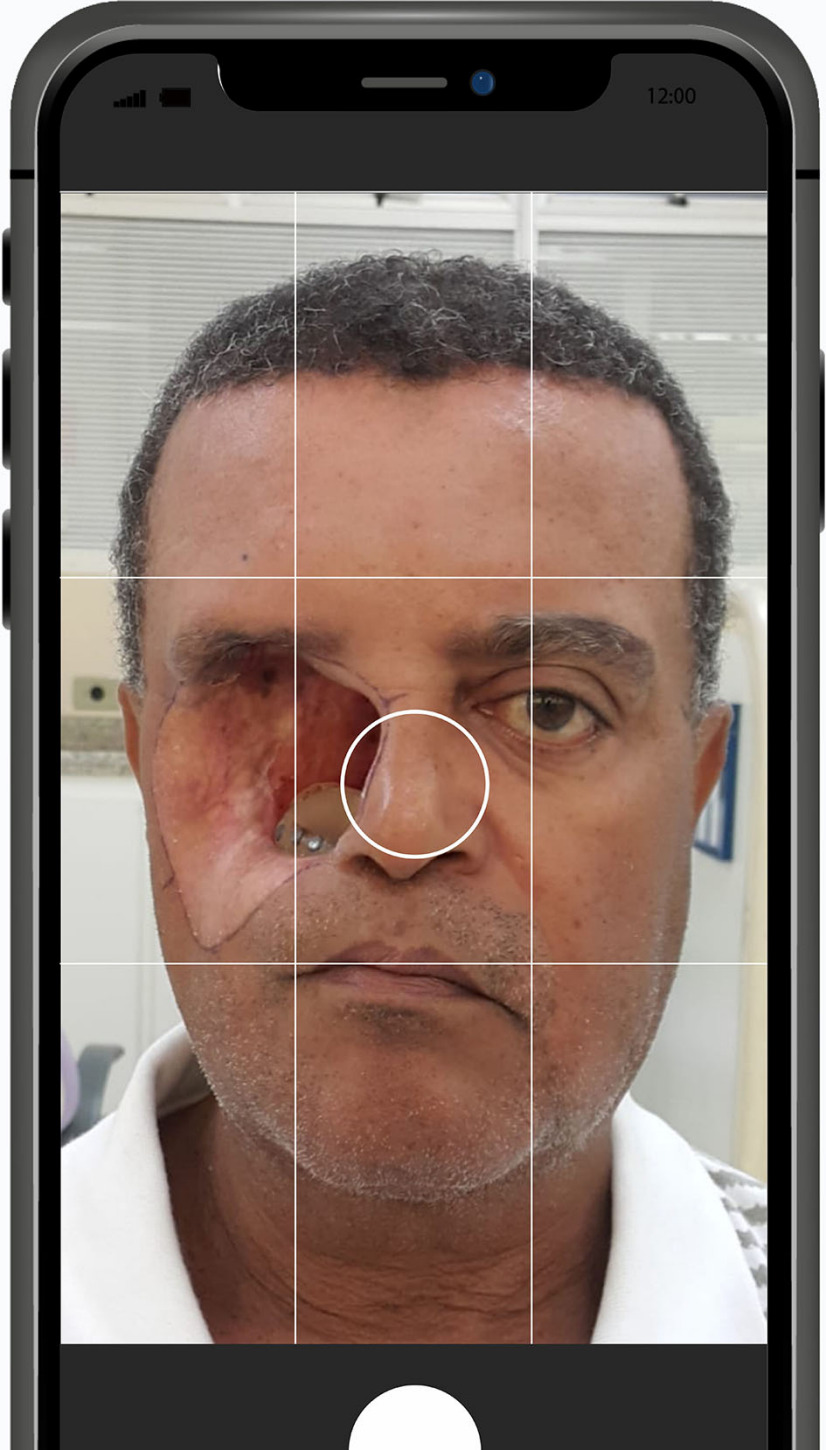
Ideal coverage of the face of the patient in each capture is approximately 80% of total image without cutting off the ears; and, in Anterior-Posterior perspective, no nose tip cutting ensuring full facial data capture. Appropriate focus is important to optimize data registration and to avoid blurry zones that may compromise the result. (Written informed consent for publication of the patient’s details and publication of the identifiable image in Figure 2 was obtained from the patient).

**Figure 3.**
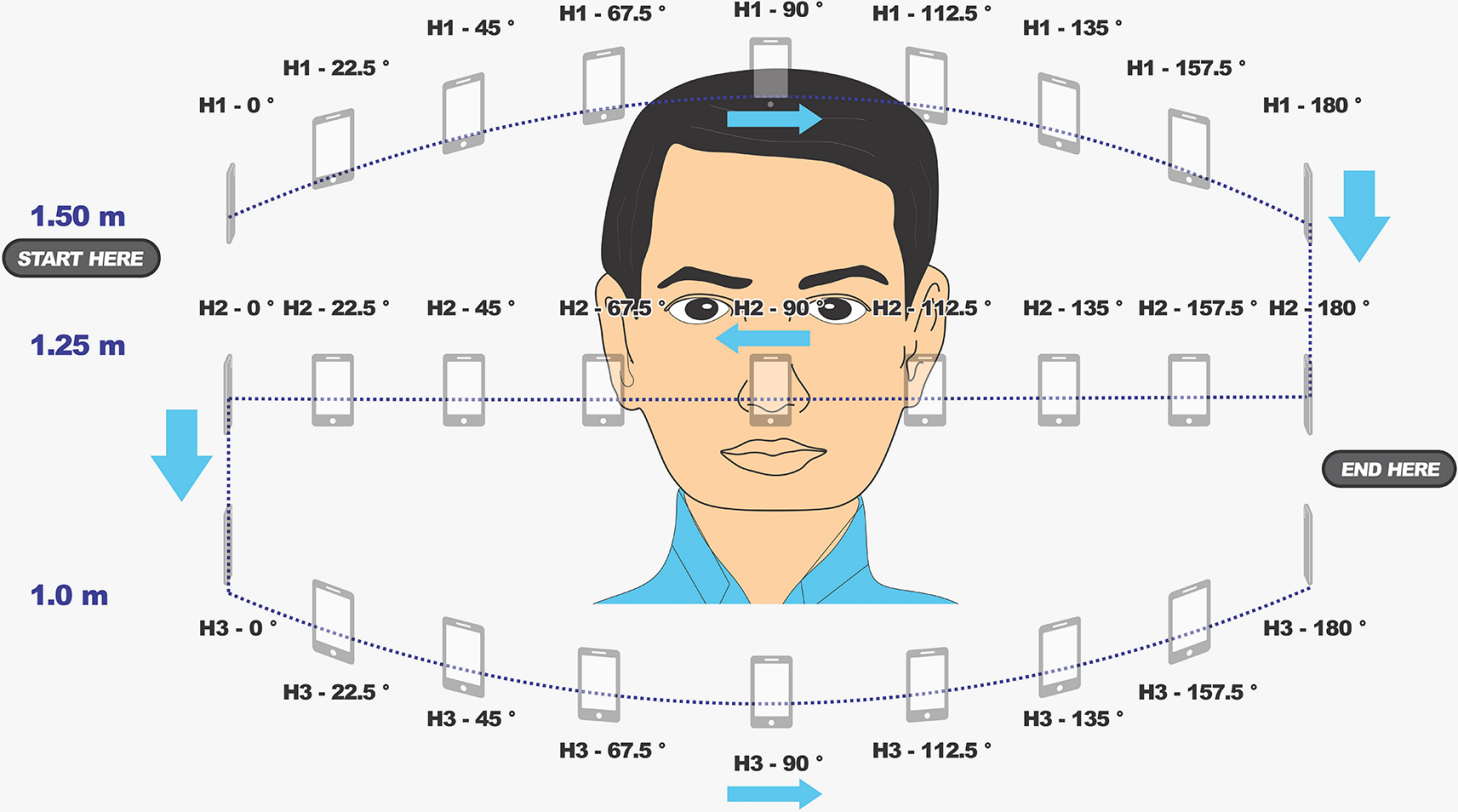
Graphic of the suggested 39 photo captures suitable for photogrammetry, according to PlusID Methodology, for an extensive orbit case like the one presented in this article. The angle between photo 1 and 13 has an angle of approximately 120 degrees with the point of interest to ensure accuracy beyond the frontal region of the face.

**Figure 4.**
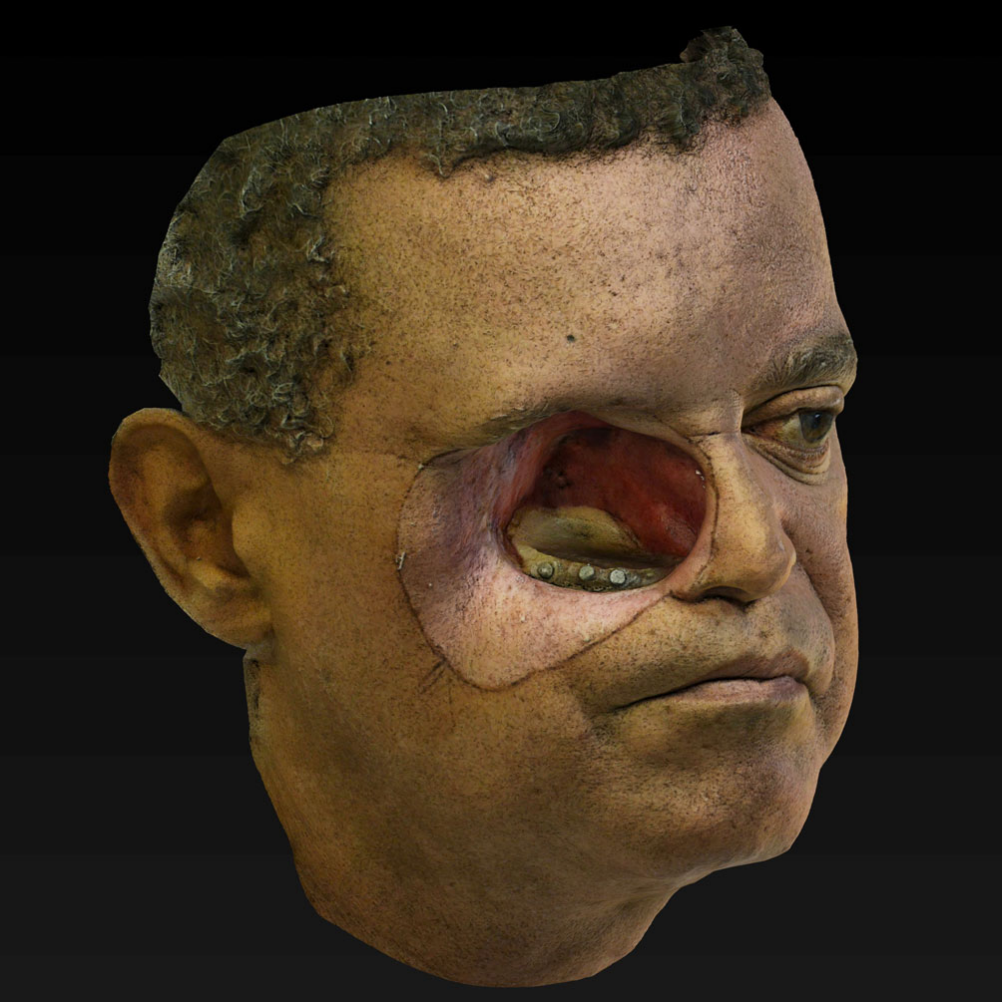
3D Model (OBJ+UVmap) of the face of our subject, obtained by the monoscopic photogrammetry feature from OrtogOnBlender after the alignment, scaling and after erasing external areas out of interest. Notice that the delimitations of the prosthesis, decided and drawn clinically before the photo captures, were appropriately reproduced and will be helpful to transport the clinical needs of the 3D modeling tools. (Written informed consent for publication of the patient’s details and publication of the identifiable image in Figure 4 was obtained from the patient).

**Figure 5.**
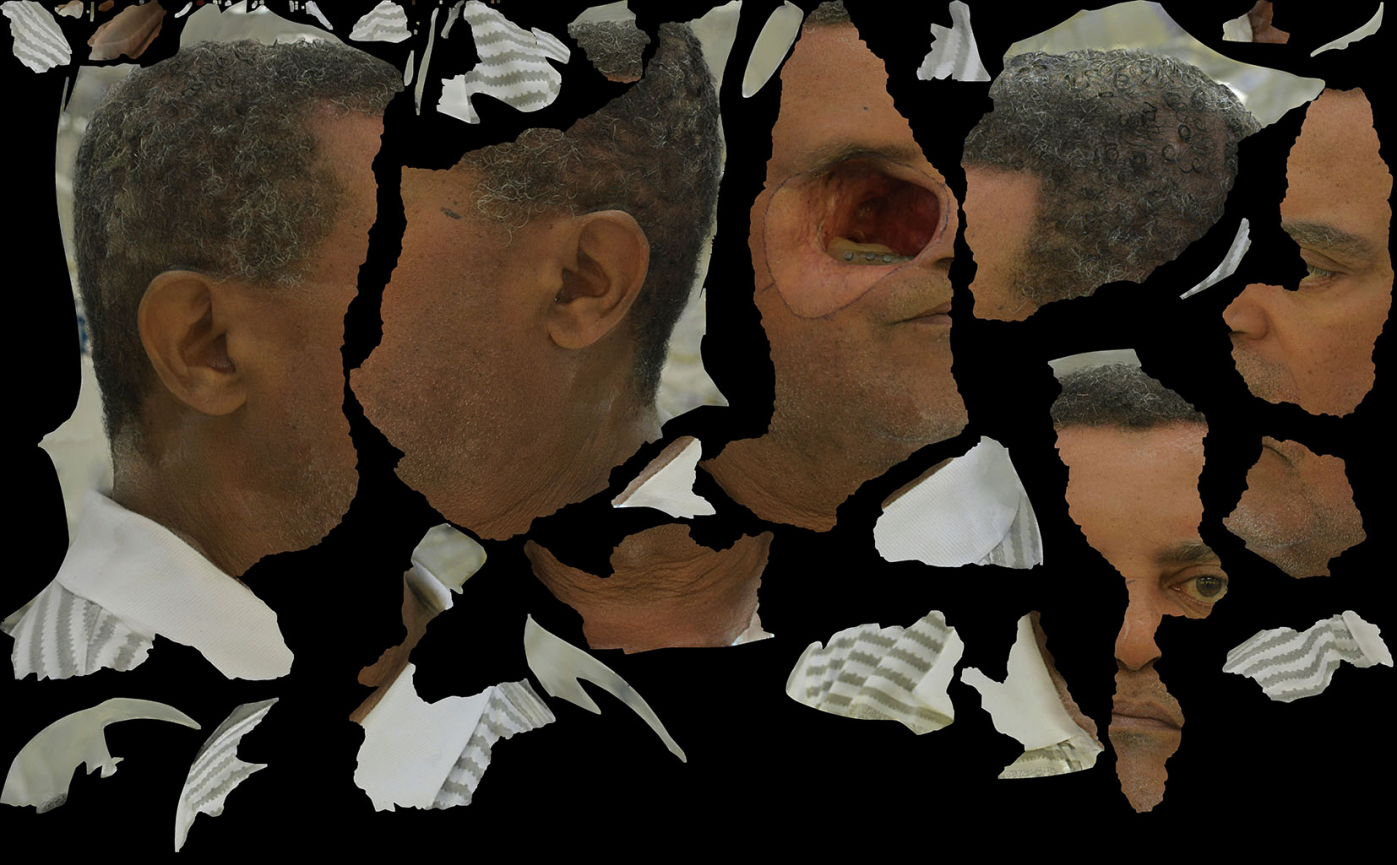
The UV-Map resultant of the photogrammetry process. This UV map wraps the 3D model (OBJ), aligned with the MTL in order to obtain full color aligned to the 3D geometry. (Written informed consent for publication of the patient’s details and publication of the identifiable image in Figure 5 was obtained from the patient).

The +ID Methodology was used to obtain the 3D model of the facial prosthesis. (The +ID Methodology refers to the combined use of smartphones and open-source software features to produce the desired 3D model of the prosthesis.) After clinical and functional evaluation, a delimitation of the margins of the prosthesis were defined, joined by a clinical measurement of the largest diameter of the area, performed with an electronic caliper for further scaling of the model ( Figure 1). No less than 1000 lux was used to illuminate the subject by indirect natural light in the scene, measured by a smartphone (Samsung Galaxy Note 8) app (Light Meter – Lux & Kelvin, Trajkovski Labs). No part of the anatomy of the face was cut out from the captures (the ears and the tip of the nose must be included). The coverage of the face of the patient was no less than approximately 80% of the screen. This “close-up” display determined the distance between the camera and the subject, focusing on the desired anatomical region; the more face coverage, the more data available for registration with each capture ( Figure 2). Optical deformations were compensated for mathematically by orthographic projections in the open-source software for 3D modeling ( OrtogOnBlender v2.80). A specific series of 39 photographs (3 different heights, 13 angles per height) were captured in this subject-operator setup ( Figure 3). A fiduciary color element (VITA-PAN ^®^ Dental Shade A2 L83 A1 B22) was placed within the capture area, a photo was then taken using the smartphone’s built-in camera, with all filters deactivated. ^16^ Files should be JPG. Captures were downloaded into a Dell computer (Model: G5 i7, 32gb ram, graphics card rtx2060) and loaded into +IDonBlender add-on to execute a monoscopic photogrammetry process selecting the OpenMVG+OpenMVS photogrammetry option, with D factor in 6 and Smooth factor at 16. After ten minutes on a computer with optimized features (i7 9 ^th^ Generation Intel Processor with 16Gb Ram and Geforce RTX/Nvidia graphic cards), an OBJ file (3D file) linked to the JPG file (Texture map/UV Map) of the subject’s face was obtained ^3^ ( Figures 4 and 5).

### 3D modeling

**Figure 6.**
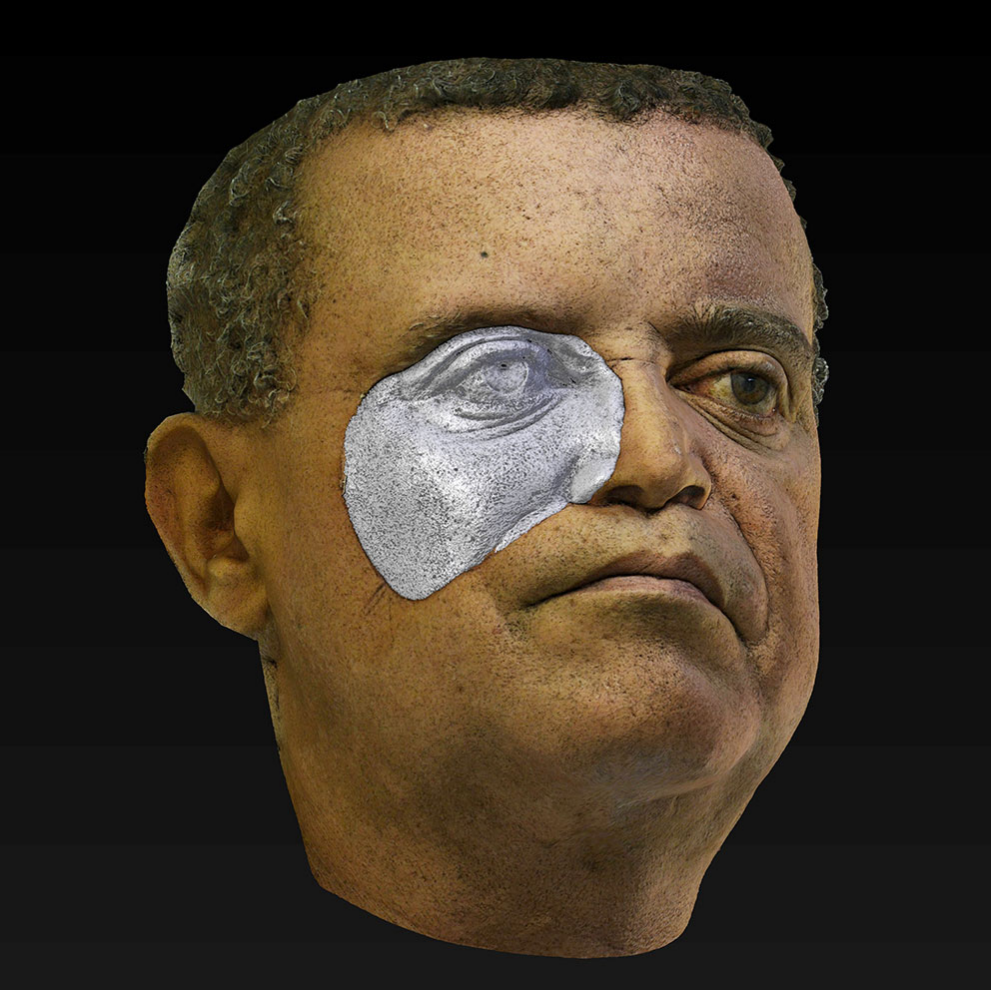
Mirroring of the healthy side of the face, sculpting tools, Boolean operations, and application of multiresolution and displacement modifiers resources were used to ensure the fit, adaptation and realistic skin detail reproduction. The prosthesis looks gray because the UV-Map was hidden, allowing the mesh to be shown. (Written informed consent for publication of the patient’s details and publication of the identifiable image in Figure 6 was obtained from the patient).

The healthy unaffected left orbit was used and mirrored over the defect ( Figure 6). 3D sculpting tools, Boolean operations, and the application of multiresolution and displacement modifiers were used to ensure reproduction of the fit, adaptation, and realistic skin detail. ^3^ The JPG of the color texture map, ^17^ including the fiduciary color element, was calibrated according to LAB color tools of Adobe Photoshop ^®^ 2020, v21.0.6, image editing software, using the fiduciary element color space coordinates as a target. The final size, form, and color of the 3D model information were exported into a VRML file, which carries information on color, transparency, brightness, texture and is compatible with the CMYKW format of 3D printers.

### 3D printing: setup, manufacture, and post-processing

**Figure 7.**
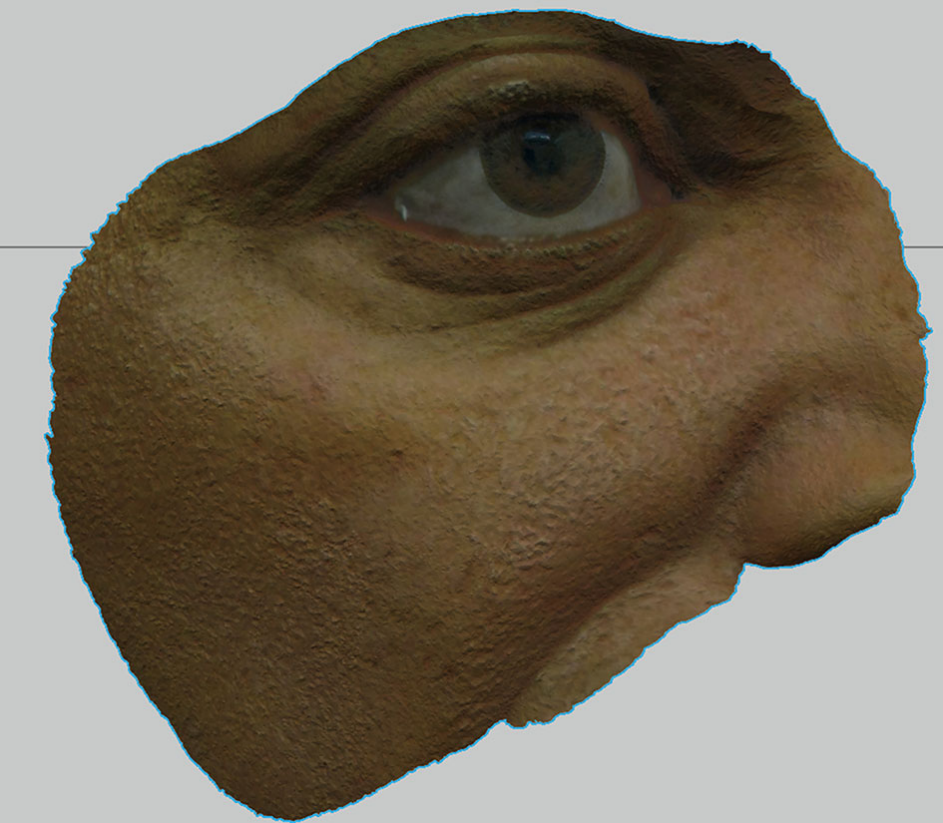
Visualization of the VRML file into GrabCad Print® software of Stratasys® suitable to communicate with the CMYKW color printing resources and with the approximation of color according to the loaded resins. (Written informed consent for publication of the patient’s details and publication of the identifiable image in Figure 7 was obtained from the patient).

**Figure 8.**
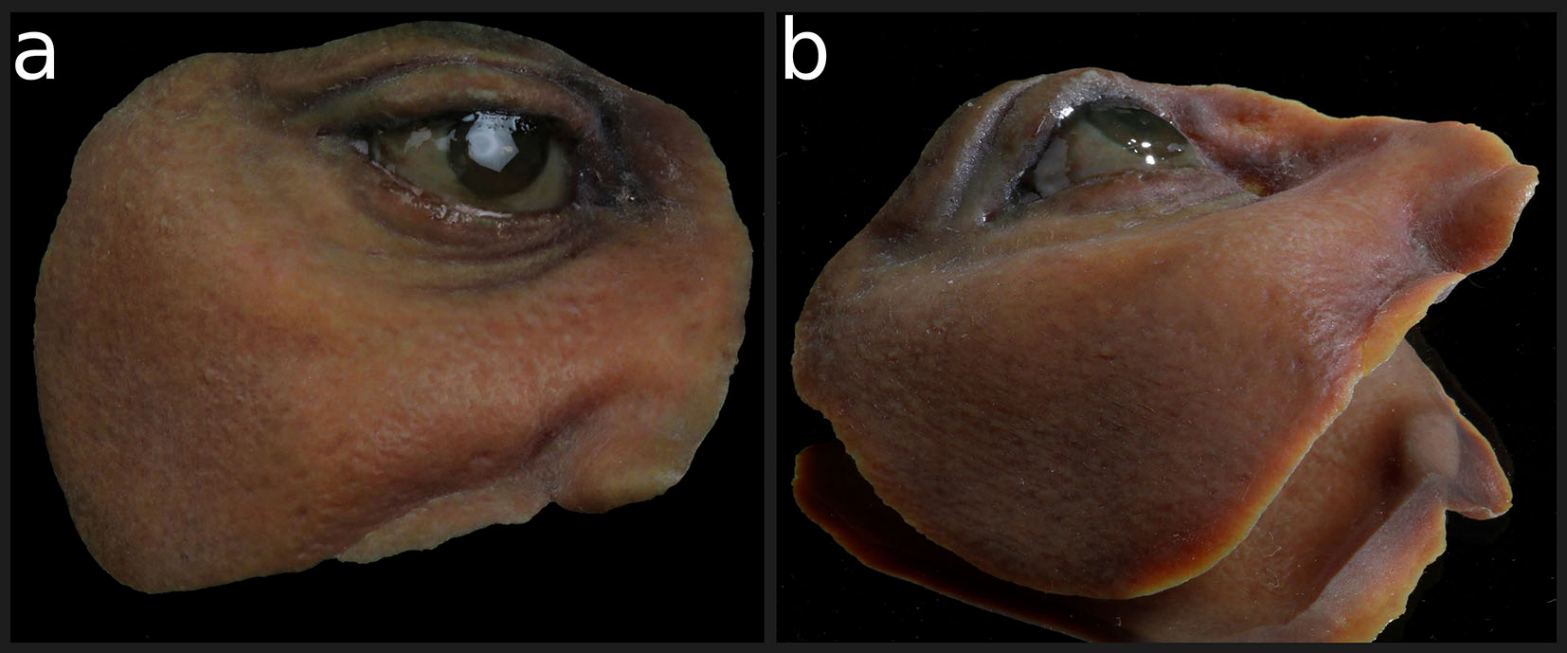
(a) Perspective of the clean 3D full colored printed facial orbital prosthesis before arrangement on the patient. Radika light cured translucent resin was applied on the eyeball structures to enhance the glaze effect. (b) Perspective of the clean 3D full color printed facial orbital prosthesis before arrangement on the patient. Radika light cured translucent resin was applied on the eyeball structure to enhance the glaze effect. (Written informed consent for publication of the patient’s details and publication of the identifiable image in Figure 8 was obtained from the patient).

The VRML file was imported into the GrabCAD Print ^®^ software v1.33 of the Stratasys ^®^ J750 3D printer. The software automatically identifies the color-per-voxel information of the VRML file and shows an approximate representation of the colors in the workbench. When selecting the color resins, the user must choose between “Absolute” or “Relative” color gamut approximation. We used “Absolute” because it creates a mathematical approximation from the RGB to the CMYKW color expression for those RGB colors out of the CMYKW gamut expression. The selected and loaded resins into the printer in this case were VeroFlexBlack, VeroFlexClear, VeroFlexCyan, VeroFlexMagenta, VeroFlexWhite, VeroFlexYellow and support resin FullCure705 ( Figure 7). Additionally, the flexible resin Agilus ^®^ was used for the base of the printed model, although due to the concentration of rigid colors, the final result was as expected, obtaining a prosthesis of high hardness. The time spent to 3D print the prosthesis was six hours and twenty-two minutes in high quality (14 microns in layer Z). The total resin used was 113 grams divided into 99 g of support resin FullCure705, 13 g of VeroFlexBlack, 13 g of VeroFlexClear, 14 g of VeroFlexCyan, 18 g of VeroFlexMagenta, 36 g of VeroFlexWhite, and 19 g of VeroFlexYellow, automatically estimated by GrabCAD Print ^®^ v1.33, using the chosen settings. The “Matte surface” was selected aiming to get a regular surface finishing for the whole part. The support material was easily removed by using some hand tools and water. The mechanism of the Polyjet 3D printer allows a layer-by-layer unique mixture of resins based on the voxel-color 3D printing, which provides an outcome of a full-color multi-material 3D printed prosthesis ( Figure 8).

### Installation

Following printing and finishing the 3D model, a few manual modifications were required in the laboratory including the manual bending of a 1 mm gap at the external right side margin adjusted with a heat gun. This was explained by the patient’s weight loss between the data acquisition process and the prosthesis installation date. Also, a slight extrinsic coloration to compensate for the color reproduction of CMYKW color expression of the 3D printer was used and afterward sealed with a thin layer of a dispersion of medical-grade Type A silicone to coat the resin prosthesis. Enhanced adhesion was obtained with a platinum primer (A304), and Bonding Enhancer (A-321) was used according to the manufacturer’s recommendations (Factor II ^®^, Lakeside AZ USA). Matting dispersion (Factor-II ^®^) was used according to manufacturer recommendations to avoid a shiny silicone surface. A thin layer of translucent photopolymerizing resin (Radika ^®^ Dentsply Sirona ^®^, Pennsylvania USA) was used to achieve the shiny surface of the ocular component.

The full-color resin 3D printed facial prosthesis was delivered on a subject with an orbital deformity. Two appointments of 20 and 45 minutes were needed to deliver the final prosthesis. Prosthetic adhesive (B-204 Pros-Aide Adhesive, Factor II) was used to attach the prosthesis to the skin. There were no concerns about weight/adhesive retention.

**Figure 9.**
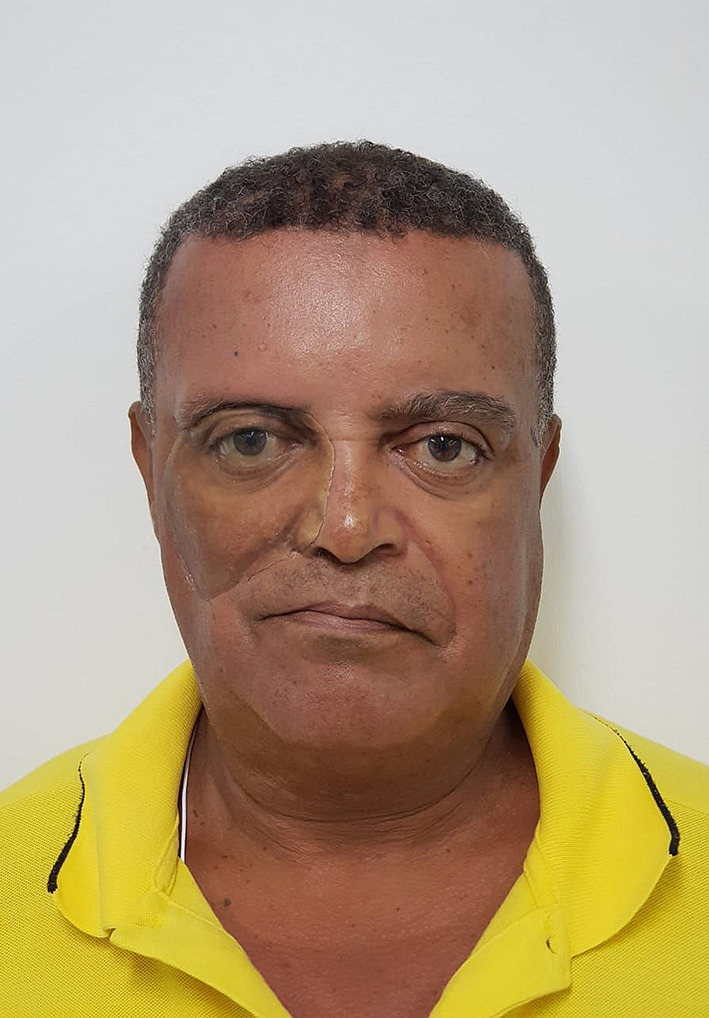
Visualization of the installed prosthesis on the patient, adhered by medical-grade prosthetic adhesive. (Written informed consent for publication of the patient’s details and publication of the identifiable image in Figure 9 was obtained from the patient).

No physical impression, direct molding, sculpture, or intrinsic silicone coloration were needed. Similarly, no separate ocular prosthesis was necessary because it was also 3D printed with the final prosthesis. No complications were observed up to the submission of this article, which was four months after prosthesis installation ( Figure 9).

## Discussion

Reproducing a patient’s skin color into a 3D printed facial prosthesis is a complex and challenging process. The manifestation of data as a physical embodiment is defined under the term “data physicalization” ^18^ or “physical visualization”. ^19^ Moreover, the physicalization of a direct 3D printed facial prosthesis has several challenges. Nevertheless, color information can be obtained by the point-based rendering of geospatial data obtained from photogrammetry methods. These visualization maps allow a user to gather insights through perception and computer-aided interaction ^20^^,^^21^ Multiple integrations of acquisition technologies, software, and digital fabrication methods have benefited facial prosthesis production around its diverse context worldwide. ^2^^,^^3^^,^^7^^,^^22^ Ultimate trending technologies have been low-cost ^1^^,^^7^^,^^16^^,^^23^^,^^24^ and accessible workflows, as well as direct 3D printing of prosthesis. ^5^^,^^13^^–^^15^

Resins for 3D printing have a different nature compared to medical grade silicone, and more studies should be conducted to understand them for this purpose. The multi-material characteristic of the latest Polyjet versions of 3D printers has much to offer to the complex composition of skin consistency and color reproduction because it can process it up to a voxel-color pigmentation, thanks to its capacity of jetting drops of approximately 40 pl (1 ^-9^ ml) each. Silicone 3D printing is evolving towards multi-material, thanks to a pre-nozzle of the silicone extruder. It was reported using multiple consistencies of silicone in one piece. ^15^ However, while the minimum drop of silicone is not close to resin, it will not be superior in the most delicate reproduction details and will depend on experienced post-processing. Silicone 3D printing demonstrated a 400-micron layer, while Polyjet resin 3D printed up to a 14-micron layer.

Both resin and silicone 3D printing systems have arguments for evolving their materials, software, and hardware technology. It will be common to see further comparisons in literature while both address their limitations. Our workflow with colorful resin 3D printing considering important technical steps is shared below to contribute to this further discussion.

### Color translation up to full-color resin 3D printing: smartphone monoscopic photogrammetry

When a photo capture is taken, the gamut of color expression is reduced from the infinite visible light expression into approximately 16.77 million colors. Here is where we start to lose color data information because any digital capture depends on an on-screen display that emits RGB (Red, Green, and Blue) light to express color. Each color channel is expressed from 0 (least saturated) to 256 (most saturated). (256 ^3^=16,777’216 colors). Captures could be saved into RAW or JPG files. JPG files are more suitable for ease of storage purposes and faster photogrammetry processing. ^16^

Color reflectance of the skin color of the subject is modified by the illumination of the ambient light. The +ID method recommends indirect frontal natural daylight. A recommended range between 1000 lux and 4000 lux will allow the automated smartphone functions of the camera to have a lower ISO, narrower diaphragm diameter, faster captures, and a better-balanced color temperature. Using a fiduciary color element and UV Map color calibration, we reduced the color discrepancy between the patient and the 3D-colored printed prosthesis. Continuous artificial light also works if it is set up closest to natural light. Manual functions of a smartphone or an SLR camera will have no better output of the 3D model and UV-map because nowadays, automatic algorithms of smartphone cameras optimize capture results. This feature also helps to reduce the micro-movements of the head and facial expressions of the patient, which, if not considered, can result in poor output.

The resultant photogrammetry 3D model (.blend) has a UV map associated and can be exported into a JPG file for further image calibration. The UV-Map has the information of the face’s color and wraps the surface of the 3D model. Once the prosthesis is 3D designed, to finally produce the 3D printing of the facial prosthesis (physical visualization), the file should be exported into a VRML file due to its compatibility with the CMYKW color 3D printing. Other printers like 3DP technology, which prints in gypsum, can print in color because its catalyzers are in CMYK tones. However, gypsum is not a suitable material for facial prosthetics and is insufficient for realistic color expression.

The color materials of the Stratasys ^®^ J750, at the time of the present publication, can materialize up to five hundred thousand colors in a 3D printed model. In contrast, conventional 2D color printing can express up to the RGB 16.77 million colors. That is why it is currently technically impossible to 2D or 3D print something precisely the same color as the actual subject’s skin. Although this technical limitation exists, the algorithms of GrabCAD Print ^®^ allow the user to choose between a Relative or Absolute approximation of the colors, aiming to get 3D printed colors that are the closest possible to the desired ones. That is why a slight layer of extrinsic coloring was applied over the external surface and fully sealed with translucent medical-grade type-A silicone. The extrinsic coloring was a mixture of orange to compensate for the greenish expression of the prosthesis of a dark skin tone of the patient. This procedure was executed to compensate for the technical limitation of the 3D printed colors to express the natural colors of our patient’s skin. This fine detailing may be understood better in further research that compares 3D printed skin color expression. Also, the possibility of using the +ID Methodology allows us to print the whole compound of the characterized eye in the same model of the whole orbital prosthesis. Thus, no separate ocular prosthesis was needed.

### Biocompatibility of 3D printed Polyjet resins

Polyjet resins have a non-toxic certification but not a biocompatibility certification from the manufacturers. Other authors faced this challenge when installing a flexible nasal prosthesis manufactured by flexible Polyjet resin in a J750 3D printer. ^6^ We considered the full coverage of the prosthesis with the medical grade type-A silicone after slight extrinsic characterization to prevent any complication. To date, the patient did not show any clinical complications, and more specific studies of Polyjet resins for this application must be conducted to guarantee patient safety in time.

### Shore

At the commencement of this research, no flexible color resin was available on the market. A high durometer prosthesis was obtained (approximately 80A Shore) due to the high concentration of pigments to achieve a darker skin color. As long as the available resins for the J750 3D printer allow white flexible resin (30A shore) but rigid (100A Shore), Cyan, Magenta, and Yellow, the darker the skin you need to reproduce the more rigid the prosthesis will become. Clearer skin colors that may need less rigid pigments for reproduction will (likely) produce a more flexible result. ^25^

In this specific case, the high durometer prosthesis was not an issue due to the almost static tissues and lack of muscular function around the limits of the patient’s prosthesis—due to post-surgical loss of nerve function following tumor resection. A high-functional muscle may present more difficulties with comfort when using a high-durometer prosthesis (darker skin). Once color resins are available, the resultant prosthesis may fit around a more appropriate range of durometers. The flexible base used was Agilus ^®^, the evolution of Tango ^®^.

### Prosthetic delivery

The Direct 3D Facial Prosthesis resulted in appropriate adaptation, approximate color matching after extrinsic coloration and was accepted by the patient who reported good comfort in wearing it. No irritation of the skin at the margins was observed up to the publication of this paper, which is four months after delivery of the prosthesis. Silicone was adhered firmly with no debonding areas up to the submission of this paper. This prosthesis was designed to be adhesively retained. The authors expect that successive designs will enable the inclusion of design options for implant retention, including designing the attachment for magnets.

### Final considerations

The manufacture of this 3D facial prosthesis depends on access to a J750 ^®^ 3D printer with the available CMYKW resins. A J750 3D printer is a professional 3D printer with special requirements about the ambient, calibration, and professionalized handling. The accessible concept is also applied when a local print center can work as a provider, with a relatively low cost of approximately $50–$150, depending on the volume and the resultant grams of resin used.

The primary purpose of 3D printing in the colorful resin Polyjet technology is the color translation of the patient tissues from the +ID Methodology. Proper computational graphic processes allow this objective, like color calibration, multiresolution and displacement, specific 3D color file exportation, and the proper setup of the printer, model, and mixture of resin loading. This alternative manufacturing method tempts to close the gap in color reproduction while the manufacturers or other 3D printing industries evolve the desired properties of the final prosthesis.

The aesthetic outcome is not yet able to replace the most skilled maxillofacial prosthodontics or anaplastologists. However, it has the chance to deliver an effortless and outstandingly fast direct colored 3D printed facial prosthesis as a temporary prosthesis or as a definitive one as determined by context. This is the crucial argument and opportunity with this workflow and its evolution: reduced working time, optimized teamwork structure, which includes diversification of technology functions to minimize reliance on individual skill-dependent capabilities, the most difficult to reproduce resource. Thus, we have the opportunity to progress toward a definitive single-step, digital, turn-key solution of facial prosthesis production, always guided by formally trained specialists.

## Conclusions

Using the +ID Methodology and full-color resin 3D printers allowed us to 3D print a resin-colored orbital final prosthesis. With no conventional molding, sculpture, and only a limited color adjustment, we obtained an acceptable adaptation and color reproduction of the final 3D prosthesis. The patient reported no complications on the usage of the prosthesis up to the time of this publication. The combined use of emerging technologies and biomaterials allows us to expand the limits of healthcare and be one step closer to a reliable, digitally driven, turn-key solution for the delivery of facial prostheses. By adapting our workflow with a more accessible learning curve, there is a more significant opportunity for more reliable provision of holistic care for patients who endure facial mutilation. With the limitations of the present study, further research is needed to determine the indications and opportunities of this digital workflow. An experienced maxillofacial prosthodontics or clinical anaplastologist, inserted into a multi-professional team, will be needed to guide design and production of a medical grade therapeutic device, and ensure appropriate rehabilitative care for the patient.

## Data availability

### Underlying data

Figshare: Underlying data for ‘Color translation from monoscopic photogrammetry +ID Methodology into a Polyjet final 3D printed facial prosthesis’. https://doi.org/10.6084/m9.figshare.19609428.v1. ^17^

Data are available under the terms of the Creative Commons Zero “No rights reserved” data waiver (CC0 1.0 Public domain dedication).

## Consent

Written informed consent for publication of the patient’s details and publication of the identifiable images in Figures 1, 2, 4, 5, 6, 7, 8 and 9 was obtained from the patient.
