# Supplementary material for: Color translation from monoscopic photogrammetry +ID Methodology into a Polyjet final 3D printed facial prosthesis
Source: F1000Res. 2022 May 27;11:582. [Version 1] doi: 10.12688/f1000research.111196.1 (PMC10904947; doi:10.12688/f1000research.111196.1)
Supplement: Peer review history

# Reviewer report: Reviewer response for version 1

**DOI:** 10.5256/f1000research.122890.r242385

**Published:** 2024-02-13

**Authors:** Frank Alifui-Segbaya

**Affiliations:** 1 Griffith University, QLD, Australia

I have reviewed the manuscript, "Color translation from monoscopic photogrammetry +ID Methodology into a Polyjet final 3D printed facial prosthesis" in which the authors present a workflow to produce a 3D printed colored prosthesis. While the scientific elements of the manuscript are minimal, the highly descriptive process could be replicated. The manuscript also contain useful information re: the limitations of using Polyjet technology for the facial prosthesis. Apart from a few typos, the manuscript is well written and contains up to date information and attempts to bridge the gap i.e., aesthetic outcomes of the age-old traditional manufacturing methods vs 3D printing.

Is the work clearly and accurately presented and does it cite the current literature?

Yes

If applicable, is the statistical analysis and its interpretation appropriate?

Not applicable

Are all the source data underlying the results available to ensure full reproducibility?

Yes

Is the study design appropriate and is the work technically sound?

Yes

Are the conclusions drawn adequately supported by the results?

Yes

Are sufficient details of methods and analysis provided to allow replication by others?

Yes

Reviewer Expertise:

3D Printing, Biomaterials, and Materials  Processing and Characterization

I confirm that I have read this submission and believe that I have an appropriate level of expertise to confirm that it is of an acceptable scientific standard.

# Reviewer report: Reviewer response for version 1

**DOI:** 10.5256/f1000research.122890.r140018

**Published:** 2022-06-30

**Authors:** Rodrigo de Faria Valle Dornelles

**Affiliations:** 1 Faculdade de Medicina da UNAERP, Universidade de Ribeirão Preto Campus Guarujá, Guarujá - SP, Brazil; 2 Universidade de São Paulo - USP, São Paulo, Brazil

In the present study, named 'Color translation from monoscopic photogrammetry +ID Methodology into a Polyjet final 3D printed facial prosthesis', the authors approach the rehabilitation of the face through the use of prosthesis in direct 3D printing. The prosthesis was designed and the production workflow described, from photogrammetric capture of surface mesh details with texturing, to hardware parameters, as well as prosthesis configuration, fabrication and post-processing.

 The study addresses a topic that has been studied so that there are alternative solutions for the rehabilitation of facial defects. Anaplastology employs techniques that are often artistic craftwork and therefore difficult to scale because it depends on skills that are not trivial. This work shows clarity in the description of technical details of a recent alternative using free software, citing the +ID method and also the introduction of possibilities of 3D printing of the prosthesis, not only to obtain the replication of the richness of details of the complex human anatomy, but also with the texturing together with the impression of the piece.

 It is essential to approach in terms of accurate printing parameters and to understand the migration of files in compatible languages ​​so that there is reproducibility of the study. It is interesting to note that there is a demonstration that the technological application of the area, essential for automation, has been sought (1), based on a review that also includes current studies (2). The possibility of using photogrammetry and the accuracy of the method for volumetric evaluation of the three-dimensional mesh has already been established in previous studies (3-4), which shows the possibility of direct adaptation due to the actual size of the prosthesis.

 When it is necessary to approach initial studies, without previous parameters, the case description is acceptable, since the innovation proposal has no parallel. The study design is adequate, as it brings the use of technology, which is always evolving with periodic updates, with the proposal to use it with current resources. Opening possibilities in the continuity of improvements, according to the appearance of new tools or materials in the area of ​​3D printing.

 Academic validity is established with the application of computer science in an area that until recently was eminently artisanal, time consuming and highly dependent on artistic subjectivity, with this, the alternative presented opens another field of approach.

 Replicating a study depends on the clarity of the methodology applied, when there is involvement of the computing area, computer configurations, such as processor, video card and RAM memory, are essential for the processes to be adequate. As well as for 3D printing parameters, even if proprietary software is used, such as the GrabCAD Print® software v1.33 of the Stratasys® J750 3D printer. This does not determine the economic viability of new projects, but it makes it possible to repeat the environment where the results were obtained and thus to be able to place new variables or even extract new data. The study described the method clearly, with details of the parameters used.

 As this is an observational, descriptive study, there was no room for statistical analysis.

 Although the capture protocol for photogrammetry had been established in a previous work (5), the variation in the number of photos captured for the present study should have been justified and substantiated. Possible changes, clarified for reasons of adequacy, will allow a better understanding of the difficulties and complexity inherent to any method, providing future collaborative work. The providential and ingenious alternative of covering the prosthesis with medical grade type-A silicone could be better described and added to the methodology, since, while there is no biocompatibility certificate from the manufacturer, other parts must undergo the same finish.

 The results presented are consistent with the conclusion that Using the +ID Methodology and full color resin 3Dprinters allowed us to 3Dprint a resin-colored orbital final prosthesis is adequate for the initial purpose of the study, which was to contribute with an innovative technology and 3D printing technique in resin with colored multimaterial. The detailing of processing care and parameters evidenced the feasibility of the method.

Is the work clearly and accurately presented and does it cite the current literature?

Yes

If applicable, is the statistical analysis and its interpretation appropriate?

Not applicable

Are all the source data underlying the results available to ensure full reproducibility?

Partly

Is the study design appropriate and is the work technically sound?

Yes

Are the conclusions drawn adequately supported by the results?

Yes

Are sufficient details of methods and analysis provided to allow replication by others?

Yes

Reviewer Expertise:

Reconstructive Plastic Surgery; Microsurgery; Cranio-maxillo-facial surgery, medical virtual modelling and 3D printing

I confirm that I have read this submission and believe that I have an appropriate level of expertise to confirm that it is of an acceptable scientific standard.

## References

[1] : Computerized color formulation for African-Canadian people requiring facial prostheses: a pilot study. *J Prosthodont* .2008;17(4) : 10.1111/j.1532-849X.2007.00288.x 327-35 10.1111/j.1532-849X.2007.00288.x 18205739

[2] : A systematic review of the computerized tools and digital techniques applied to fabricate nasal, auricular, orbital and ocular prostheses for facial defect rehabilitation. *J Stomatol Oral Maxillofac Surg* .2020;121(3) : 10.1016/j.jormas.2019.10.003 268-277 10.1016/j.jormas.2019.10.003 31610244

[3] : New virtual tool for accurate evaluation of facial volume. *Acta Cir Bras* .32(12) : 10.1590/s0102-865020170120000009 1075-1086 10.1590/s0102-865020170120000009 29319736

[4] : 3D Virtual Planning for Rhinoplasty Using a Free Add-On for Open-Source Software. *Aesthetic Surgery Journal* .2021;41(8) : 10.1093/asj/sjab085 NP1024-NP1032 10.1093/asj/sjab085 33581695

[5] : Monoscopic photogrammetry to obtain 3D models by a mobile device: a method for making facial prostheses. *J Otolaryngol Head Neck Surg* .2016;45(1) : 10.1186/s40463-016-0145-3 33 10.1186/s40463-016-0145-3 27225795 PMC4881215
